# Antibiotics from Deep-Sea Microorganisms: Current Discoveries and Perspectives

**DOI:** 10.3390/md16100355

**Published:** 2018-09-29

**Authors:** Emiliana Tortorella, Pietro Tedesco, Fortunato Palma Esposito, Grant Garren January, Renato Fani, Marcel Jaspars, Donatella de Pascale

**Affiliations:** 1 Institute of Protein Biochemistry, National Research Council, I-80131 Naples, Italy; e.tortorella@ibp.cnr.it (E.T.); tedesco@insa-toulouse.fr (P.T.); f.palma@ibp.cnr.it (F.P.E.); g.january@ibp.cnr.it (G.G.J.); 2 Laboratoire d’Ingénierie des Systèmes Biologiques et des Procédés, INSA, 31400 Toulouse, France; 3 Stazione Zoologica “Anthon Dorn”, Villa Comunale, I-80121 Naples, Italy; 4 Department of Biology, University of Florence, Sesto Fiorentino, I-50019 Florence, Italy; renato.fani@unifi.it; 5 Marine Biodiscovery Centre, Department of Chemistry, University of Aberdeen, Aberdeen, Scotland AB24 3UE, UK; m.jaspars@abdn.ac.uk

**Keywords:** antibiotics, deep-sea, marine microorganisms, extreme habitat, marine sediments

## Abstract

The increasing emergence of new forms of multidrug resistance among human pathogenic bacteria, coupled with the consequent increase of infectious diseases, urgently requires the discovery and development of novel antimicrobial drugs with new modes of action. Most of the antibiotics currently available on the market were obtained from terrestrial organisms or derived semisynthetically from fermentation products. The isolation of microorganisms from previously unexplored habitats may lead to the discovery of lead structures with antibiotic activity. The deep-sea environment is a unique habitat, and deep-sea microorganisms, because of their adaptation to this extreme environment, have the potential to produce novel secondary metabolites with potent biological activities. This review covers novel antibiotics isolated from deep-sea microorganisms. The chemical classes of the compounds, their bioactivities, and the sources of organisms are outlined. Furthermore, the authors report recent advances in techniques and strategies for the exploitation of deep-sea microorganisms.

## 1. Introduction

The deep-sea is one of the less explored and extreme environments on Earth [1]. The characteristics of the deep-sea that make it an extreme environment include (i) pressure increases by one atmosphere (atm) for every 10-m increase in water depth, so pressure varies from 20 atm at the shelf-slope break to >1000 atm in the deepest parts of the trenches; (ii) temperature generally drops with increasing depth reaching values around 2 °C on the abyssal plain; (iii) the oxygen concentration in the bottom waters can be much less than that of the surrounding region, or even zero, depending on the balance between the rate at which oxygen is supplied (by exchange with the atmosphere and as a byproduct of photosynthesis by marine plants in the euphotic zone) and the rate at which it is consumed; and (iv) light intensity declines exponentially with depth in the water column because incident photons are absorbed or scattered, and total darkness prevails below 250 m deep [2]. The definition of deep-sea environment is still variable. Older designations describe the deep-sea as having a depth above 200 m, but more commonly, it is considered a depth ≤1000 m as standard. For many years, the difficulty to reach the bottom of the ocean has been the main issue for studying deep-sea life. Recently, thanks to the improved acoustic technology and the improved access by Remotely Operated Vehicles (ROV) and submersibles, deep ocean environments became more accessible, unveiling the presence of biological activity [3]. The access to deep-sea organic material combined with the application of culture-dependent and -independent methods demonstrated the presence of an unexpected microbial biodiversity [4,5,6]. Microorganisms inhabiting these harsh environments developed unique strategies to survive, especially to the high pressure. In fact, most of them are piezotolerant and piezophilic microorganisms [7], but the lack of appropriate instrumentation limits the cultivation of these strains. Their adaptation to biochemical and physiological processes is mirrored in modifications to gene regulation and primary/secondary metabolic pathways that result in the expression of novel natural products (NPs).

In the last 50 years, over 30,000 marine natural products (NPs) have been found and approximately 2% of those have been isolated from deep-sea organisms [8]. Among microbial NPs, antibiotics are one of the most interesting molecules, especially for biotechnological and pharmaceutical applications. The discovery of novel antibiotics is necessary to counteract the spread of multidrug-resistant (MDR) bacteria [9], and the exploitation of unexplored deep-sea microorganisms, such as bacteria and fungi, and their related products, could lead to the isolation of new antibiotics. 

This review reports on novel bioactive compounds with antibiotic activity isolated from deep-sea bacteria and fungi. We also provide an update on the current state-of-the-art of deep-sea bioprospecting, discussing bottlenecks and current advances in the field, from sampling techniques and cultivation to metagenomic approaches.

## 2. Antimicrobial Compounds from Marine Microorganisms

### 2.1. Antimicrobial Compounds from Bacteria

**Figure 1:**
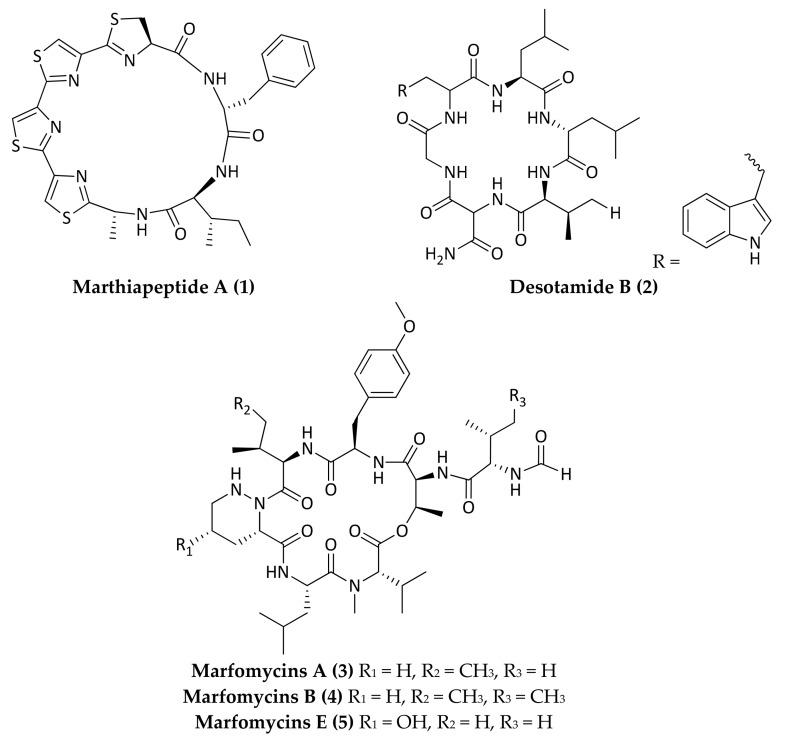
Molecular structure of new cyclic peptides with antimicrobial activity isolated from deep-sea bacteria. Compound (**1**), marthiapeptide A, is a polythiazole cyclic peptide isolated from *M. thermotolerans* SCSIO 00652. Compound (**2**), desotamide B, is a cyclic hexapeptide isolated from *Streptomyces scopuliridis* SCSIO ZJ46. Compounds (**3**–**5**), marfomycins A, B, and E, are cyclic hepta-depsipeptides isolated from *Streptomyces drozdowiczii* SCSIO 10141.

**Figure 2:**
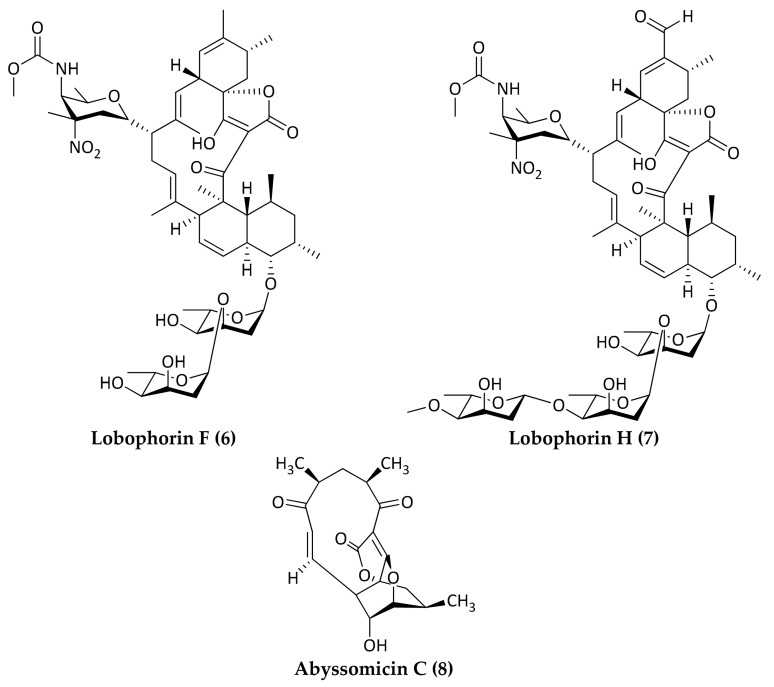
Structures of new spirotetronate polyketides with antimicrobial activities isolated from deep-sea bacteria. Lobophorin F (**6**) was isolated from *Streptomyces* SCSIO 01127. Lobophorin H (**7**) was isolated from *Streptomyces* sp. 12A35*S*; Abyssomicin C (**8**) was isolated from *Verrucosispora* strain AB 18-032.

**Figure 3:**
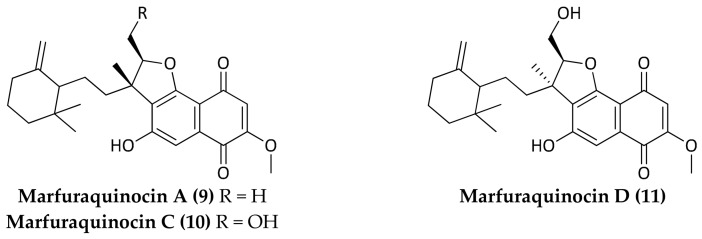
Structures of Marfuraquinocins A (**9**), C (**10**), and D (**11**) isolated from *Streptomyces niveus* SCSIO 3406.

**Figure 4:**
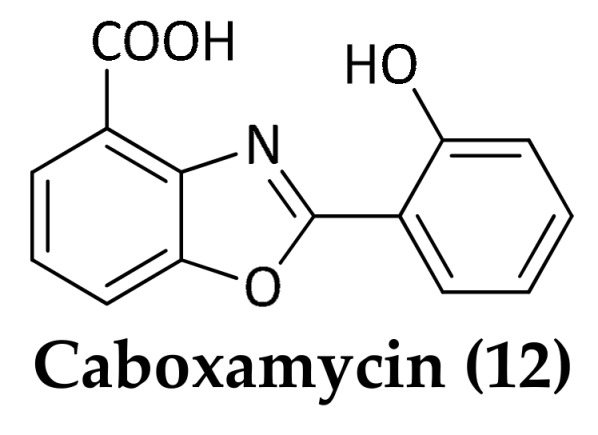
Structure of caboxamycin (**12**).

**Figure 5:**
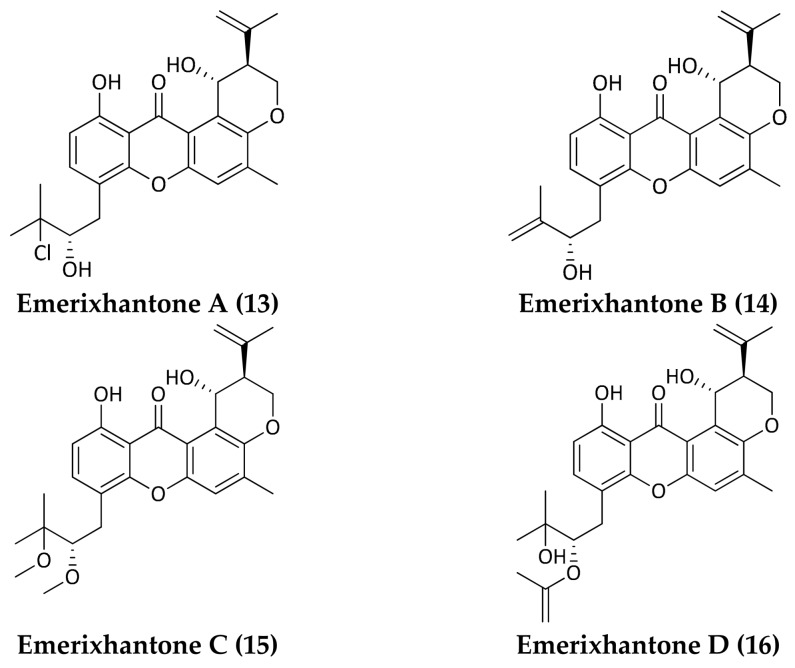
Structures of Emerixanthones A–D (**13**–**16**).

**Figure 6:**
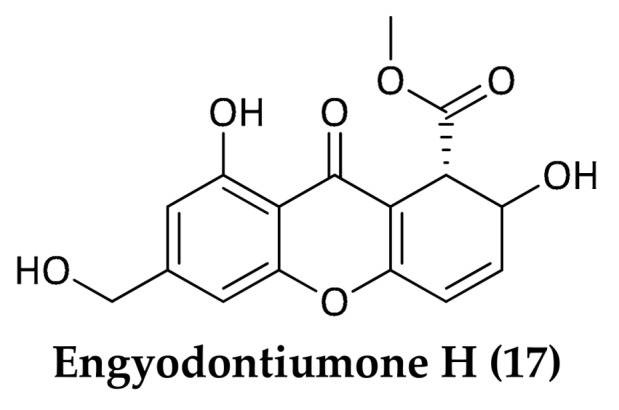
Structure of Engyontiumone H (**17**).

**Figure 7:**
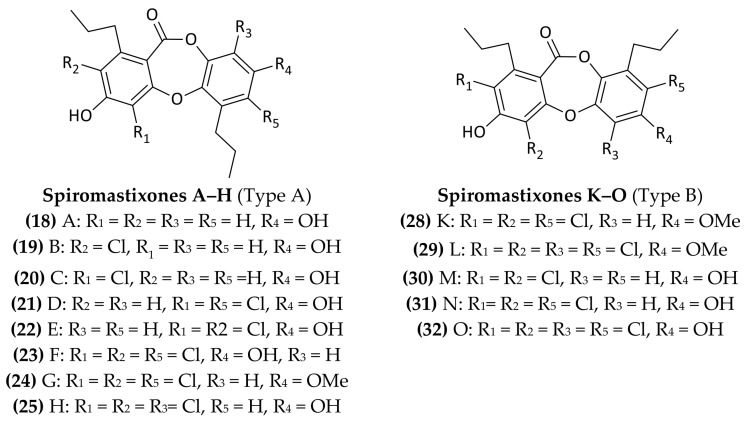
Structures of Spiromastixones A–O (**18**–**32**).

**Figure 8:**
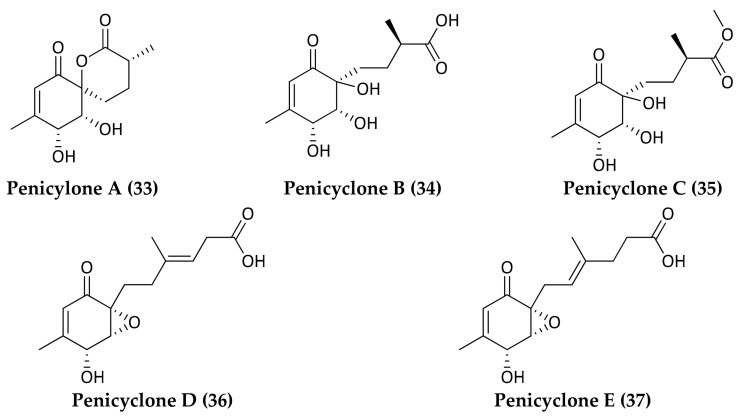
Structures of Penicyclones A–E (**33**–**37**).

Marine microorganisms represent a significant source for the discovery and development of new antibiotics due to their rich biodiversity and genetic capacity to produce unique metabolites. It is recognized that many taxonomically novel species are promising sources of new bioactive compounds [10]. In particular, marine bacteria derived from deep-sea sediments have shown to be a rich source of secondary metabolites with novel structures and excellent biological activities, including antimicrobials [11,12,13,14,15]. Most natural antibiotics are biosynthesized by bacteria belonging to the high GC Gram-positive bacteria. In particular, actinomycetes represent the most important source of bioactive natural products with clinical or pharmaceutical applications [16]. Most of the antimicrobial compounds shown below (Compounds **1**–**12**) were isolated from deep-sea derived actinomycetes (Figure 1, Figure 2, Figure 3, Figure 4, Figure 5, Figure 6, Figure 7 and Figure 8; Table 1).

Tian et al. first discovered and classified the type-strain of the novel marine actinomycete, *Marinactinospora thermotolerans* SCSIO 00652, isolated from a sediment collected from site E410 (1 °58.742′ N 11 °00.228′ E; black soft mud at 3865 m depth) in the northern South China Sea [17]. Thereafter, Zhou et al. purified a new polythiazole cyclic peptide, referred to as marthiapeptide A (**1**), from this organism. Marthiapeptide A (**1**) exhibited strong antibacterial activity against *Micrococcus luteus*, *Staphylococcus aureus* ATCC 29213, *Bacillus subtilis* ATCC 6633, and *B*. *thuringiensis*, with MIC values of 2, 8, 4, and 2 µg/mL, respectively [18]. Three new cyclic hexapeptides, named desotamides B, C, and D were identified from *Streptomyces scopuliridis* strain SCSIO ZJ46, which was isolated from a South China Sea sample sediment collection at a depth of 3536 m (120°0.250′ E, 20°22.971′ N). Among the new compounds identified, desotamide B (**2**) showed an antimicrobial activity against *S. aureus* ATCC 29213, *Streptoccocus pneumoniae* NCTC 7466, and methicillin-resistant *Staphylococcus epidermidis* (MRSE) shhs-E1, with MIC values of 16, 12.5, and 32 µg/mL, respectively [19]. New cyclic congeners, marfomycins A (**3**), B (**4**), and E (**5**), were isolated from the South China Sea-derived *Streptomyces drozdowiczii* SCSIO 10141. The producer strain of marfomycins A, B, and E was isolated from a sediment collected at a depth of 1396 m (118°58.2475′ E, 22°2.3689′ N) [20]. Unique *N*-terminally formylated side chain and five nonproteinogenic amino acid residues characterize marfomycins A (**3**), B (**4**), and E (**5**). Marfomycins A (**3**), B (**4**), and E (**5**), exhibited a selective anti-infective activity against *M. luteus*, among a panel of Gram-positive and Gram-negative bacteria, with MIC values of 0.25, 4, and 4 µg/mL, respectively. The antimicrobial agents marthiapeptide A (**1**), desotamides B (**2**), and marfomycins A (**3**), B (**4**), and E (**5**) (Figure 1) represent new deep-sea derived cyclic peptides. Cyclic peptides and depsipeptides are secondary metabolites of microorganisms and plants, with a recognized broad spectrum of biological properties [21,22,23]. This class of NP represents a valuable source for the discovery of new therapeutics, due to their favorable properties, such as resistance to enzymatic degradation [24]; a large surface area, which provides high affinity and selectivity for the targets [25]; and limited conformational flexibility [26,27], which enhances binding properties. Moreover, they often possess better membrane penetration properties [28]. As a result, they have much longer half-lives in vivo than their acyclic counterparts, and are thus of great interest in the field of drug discovery [29]. Cyclic antimicrobial peptides (AMPs) have emerged as good antimicrobial candidates due to their aforementioned characteristics [21,22,23,24,25,26,27,28,29] and high activity [30,31]. The molecular details of the action of cyclic AMPs, that is, whether these short peptides can open and stabilize pores, are still unclear, as well as the molecular basis for their antibacterial activity.

Another family of microbial metabolites with potent antitumor and antibiotic properties is collectively designated as spirotetronate polyketides. Three new spirotetronate polyketides with antibacterial activity isolated from deep-sea Gram-positive bacteria are shown in Figure 2.

Two different research groups identified the already-known lobophorins B and two new spirotetronate antibiotics, designated lobophorins F (**6**) and H (**7**), from actinomycetes isolated from deep-sea sediments of the South China Sea [32]. Both lobophorins F (**6**) and H (**7**) were extracted from *Streptomyces* sp. strains (SCSIO 01127 and 12A35, respectively). The two strains were isolated from a sediment sample collected at the depth of 1350 m (111°54.693′ E, 08°56.003′ N) [33] and 2134 m (17°59.928′ N, 111°36.160′ E), respectively [34]. Lobophorin F (6) showed antibacterial activity against *S. aureus* ATCC 29213 and *Enterococcus faecalis* ATCC 29212 with MIC values of 8 µg/mL for both of the strains. Lobophorin H (**7**) showed antibacterial activity against *B. subtilis* CMCC63501 with a MIC value of 3.13 µg/mL. Based on the inhibitory activity exhibited against Gram-positive bacteria, lobophorins F (**6**) and H (**7**) may potentially find application in antibacterial drug development. Moreover, the discovery of more spirotetronate antibiotics analogs helps to elucidate the structure–activity relationships and potential applications of these compounds. Lobophorin F and H are analogs of lobophorin B, which was previously isolated from an alga-associated actinobacterium; lobophorin B is structurally related to another spirotetronate antibiotic, named kijanimicin [35]. Recent studies indicate that kijanimicin binds to the TetR family of transcriptional regulators that control the expression of various cytoplasmic proteins in prokaryotes [36].

Bister et al. isolated three new polycyclic polyketide-type antibiotics, named abyssomicins B, C, and D from the rare actinomycete *Verrucosispora* strain AB 18-032, which originated from a sediment sample collected in the Japanese Sea at a depth of 289 m [37]. Abyssomicin C (**8**) showed antibiotic activity against methicillin-resistant *S. aureus* (MRSA) and a vancomycin-resistant *S. aureus* strain with MIC values of 4 µg/mL and 13 µg/mL, respectively. Abyssomicin C is considered a representative member of class I spirotetronate. Abyssomicin C and its atropisomer inhibit para-aminobenzoic acid (pABA) biosynthesis, these compounds are the first known substances derived from a bacterial source that inhibit the biosynthesis of pABA. The biosynthesis of pABA is an attractive target in the field of new antibiotics discovery since it is found in many microorganisms but not in humans, and blocking the pABA pathway damages bacteria since it is a biosynthetic precursor of folic acid, which is essential for DNA synthesis/repair and cell survival [35].

In 2013, three new sesquiterpene derivatives, named marfuraquinocins A (**9**), C (**10**), and D (**11**), were purified from the fermentation broth of *Streptomyces niveus* SCSIO 3406 isolated from a South China Sea sample sediment (120°0.250′ E, 20°22.971′ N) obtained from a depth of 3536 m [38]. Marfuraquinocins A (**9**), C (**10**), and D (**11**) (Figure 3) exhibited antibacterial activity against *S. aureus* ATCC 29213 with equivalent MIC values of 8 µg/mL; moreover marfuraquinocins C (**10**) and D (**11**) showed antibacterial activity against methicillin-resistant *S. epidermidis* (MRSE) shhs-E1 with MIC values of 8 µg/mL. 

Hohmann et al. detected caboxamycin (**12**) (Figure 4), a new antibiotic of the benzoxazole family, in extracts of the strain *Streptomyces* sp. NTK 937 originated from an Atlantic Ocean deep-sea sediment (27°02′392 N, 18°29′022 W) at a depth of 3814 m [39]. Caboxamycin showed inhibitory activity against the Gram-positive bacteria *B. subtilis* (IC_50_ = 8 µM) and *Staphylococcus lentus* (IC_50_ = 20 µM) and the opportunistic pathogen *S. epidermidis* (IC_50_ = 43 µm). Notable molecular targets of caboxamycin are phosphodiesterases, which are essential regulators of cyclic nucleotide signaling with diverse physiological functions.

### 2.2. Antimicrobial Compounds from Fungi

Since the discovery of the antibiotic cephalosporin in 1948, marine fungi have been considered an excellent source of bioactive compounds. Thanks to their unique adaptive capabilities, marine fungi are able to colonize different marine habitats, even the most extreme ones, including deep-sea environments. Although several studies have reported that fungi are abundant and diverse in these habitats [40], it is anticipated that many remain to be discovered. Access to the actual fungal biodiversity present in the deep-sea could lead to the discovery of new bioactive compounds useful for drug discovery [41]. The first antimicrobial compound isolated from an *Aspergillus* sp. strain originating from deep-sea sediments was gliotoxin, which is able to inhibit the growth of the Gram-positive bacteria *S. aureus* and *B. subtilis* [42]. The fungus was isolated from the mud of Seto Inland Sea in Japan. Subsequent to this discovery, other new antibiotics were detected from deep-sea fungi but they still represent a minority compared to molecules produced by marine fungi isolated from surface waters [43]. Zhang et al. [44] isolated 13 novel fungal species (among them some new phylotypes) from deep-sea sediments in the South China Sea and many of them were able to produce antimicrobial compounds against pathogenic bacteria and fungi, like *Micrococcus luteus*, *Pseudoaltermonas piscida, Aspergerillus versicolor*, and *A. sydowii* [44].

Prenylxanthones are an important group of naturally occurring secondary metabolites endowed with a wide range of biological and pharmacological activities [45,46]. Four new antifungal and antibacterial prenylxanthones, emerixanthones A–D (**13**–**16**) (Figure 5), were identified from the deep-sea fungus *Emericella* sp. SCSIO 05240, isolated in the South China Sea (3258 m). These molecules are able to inhibit *Escherichia coli* (ATCC 29922), *Klebsiella pneumoniae* (ATCC 13883), *S. aureus* (ATCC 29213), *E. faecalis* (ATCC 29212), *Acinetobacter baumannii* (ATCC 19606), and *Aeromonas hydrophila* (ATCC 7966) [47]. Another compound belonging to the xanthone class, engyodontiumone H (**17**) (Figure 6), was purified from *Engyodontium album* DFFSCS021, a deep-sea fungus collected at 3739 m depth in the South China Sea. This molecule exhibited inhibitory activity against *E. coli* and *B. subtilis* with MIC values of 64 μg/mL and 32 μg/mL, respectively [48]. A study carried out by Huang et al. showed that xanthone derivatives could act as antibiotics by blocking the enzyme I (EI) of the bacterial phosphoenolpyruvate-dependent phosphotransferase system (PTS) [49].

Fifteen new depsidone-based analogs, spiromastixones A–O (**18**–**32**) (Figure 7), have been isolated from an unidentified *Spiromastix* sp. fungus, collected at a depth of 2869 m in the South Atlantic Ocean. These compounds showed an antimicrobial activity towards *S. aureus*, *B. thuringiensis*, and *B. subtilis* with MIC values ranging from 0.125 to 8.0 μg/mL. Moreover, some of them displayed inhibitory effects on methicillin-resistant strains of both *S. aureus* (MRSA) and *S. epidermidis* (MRSE), and also against vancomycin-resistant *E. faecalis* and *E*. *faecium* (VRE) strains [50]. 

New chlorinated azaphilone pigments showing antibacterial and cytotoxic activities have been recently identified from a deep-sea fungus *Chaetomium* sp. strain NA-S01-R1, isolated at a depth of 4050 m in the West Pacific Ocean [51].

Several analyses of fungal genomes demonstrated that the number of predicted biosynthetic genes exceeds the number of new molecules obtained so far, leaving many compounds yet to be discovered [52]. The application of the OSMAC approach (one strain many compounds) could stimulate the expression of cryptic genes leading to the identification of novel antibiotics as demonstrated by Wenqiang Guo et al. [53]. This study reported that a deep-sea-derived fungus *Penicillium* sp. F23-2, collected from Jiaozhou Bay in China at a depth of 5080 m [54], produced five new ambuic acid analogs, penicyclones A−E (**33**–**37**), which exhibited antimicrobial activity against *S. aureus* with MIC values ranging from 0.3 to 1.0 μg/mL. This fungus showed a different metabolites production when grown on rice-based medium instead of PYG and PD media [53]. Improving isolation methods and exploring different cultivation approaches of deep-sea fungi could become an important weapon against multidrug-resistant bacteria.

## 3. Improving the Biodiscovery Pipeline for Deep-Sea Antibiotics

As shown in Section 2, several antimicrobial compounds have been discovered thus far from deep-sea environments. Nonetheless, we are very likely scratching the surface of a wider reservoir of molecular scaffolds. The discovery of novel antibiotics is per se linked to the discovery of novel biological resources (microorganisms and/or their genetic material) but there are still many bottlenecks limiting the success of biodiscovery campaigns for antimicrobials, and more generally for bioactive compounds, from this extreme ecosystem(s). To improve the current situation, innovations aimed at realizing the exploitation of deep-sea microorganisms are essential. In this section, we deal with the major limitations for deep-sea microbial investigations and report current knowledge and innovations available to researchers.

### 3.1. Sampling Techniques

Marine sediments experience extremely high pressures, with approximately 58% of the seafloor’s surface being at of 4000 m and above, with up to 40 MPa of pressure. One of the major limitations of deep-sea sampling for bacteria isolation is that most of microorganisms inhabiting these sites are obligate piezophiles, unable to grow at atmospheric pressure but especially sensitive to shifts in pressure [55,56,57,58]. Hence, pressurized transport and treatment of sub-sea floor sediments to the surface is a crucial step to preserve microbial viability and diversity. In 2002, the German Project OMEGA developed a Multiple Autoclave Corer (MAC) able to collect four 0.6 m cores maintaining in situ conditions (e.g., pressure and temperature) for geological and microbiological purposes. The cores, collected at −776 m in the North Pacific Ocean, were still pressurized after two months [59]. Further efforts developed a Dynamic Autoclave Piston Corer (DAPC) designed for a maximum water depth of 2000 m. The research group of Parkes and colleagues established a complete system, which includes a ‘hydrate autoclave coring equipment’ (HYACE), a pressurized core subsampling and extrusion system (PRESS), and pressurized chambers for prokaryotic enrichment and isolation (DeepIsoBUG) [60]. After the core is taken from the sea-floor and brought on the vessel without depressurization (up to 25 MPa), it passes to subcoring and slicing system to obtain a sample subcore (20 mm) that is further sliced. The slice is then transferred to a low-pressure vessel (max 25 MPa), which, through shaking, produces a slurry that can be used as an inoculum. The slurry is then transferred to high-pressure culture vessels at different pressures (up to 100 Mpa) containing enrichment media. The isolation chamber has 12 agar plates attached to a motor-driven chain, to select individual plates [60]. Thus, sediments never experience depressurization, helping to maintain the integrity and the value of the samples. The most recent development in deep-sea sampling involves utilization of robots, which is defined as “Soft robotic grippers” [61]. Galloway et al. have built a remotely operated vehicle (ROV) equipped with a robotic-hand able to delicately handle marine microorganisms. In their pilot study, the Deep Reef ROV and the soft robotic grippers were brought to the Gulf of Eilat in the northern Red Sea. The device was employed at depths between 100 and 170 m and proved to be able to grab soft specimen without damage. Despite its current limitations (it can operate at limited depths and only with macroorganisms), this device could significantly help in the study and exploration of the deep-sea. Future developments, as foreseen by Galloway, can include technical modification to add complete robotic-hands and perform experiments underwater, and the possibility to add RNA preservers (e.g., RNAlater) to facilitate transcriptomic experiments. This can aid the research on marine symbionts, known producers of metabolites, and help in the discovery of novel genes involved in the production of antimicrobial compounds.

### 3.2. Isolation and Cultivation Techniques

Isolation and cultivation of deep-sea microorganisms started in the late 1950s, when Zobell and Morita, true pioneers in this field, succeeded in the development of titanium vessels for bacterial growth that could reach 100 MPa of pressure. They were not able to isolate obligate piezophilic bacteria but only piezotolerant strains [62].

To obtain the first obligate piezophilic strains, it was necessary to wait until 1979, when Yayanos et al. were able to isolate spirillium CNPT-3 from an amphipod collected in Philippines’ trenches using a pressure-retaining trap [63].

In the following years, many piezophiles have been isolated, but the majority of them represented few bacterial taxa and did not reflect the extent of deep-sea biodiversity [64,65].

Classical isolation methods require enrichment steps and nutrient-rich media, which often retrieve dominant fast-growing taxa. Innovative isolation techniques have been used to isolate novel strains such as using a dilution-to-extinction cultivation method employing a natural seawater medium to obtain a novel member of *Roseobacter* clade within alphaproteobacteria [66]. The strain, named PRT1, is an obligate psychropiezophile and resulted in being the slowest-growing (minimal doubling time, 36 h) and lowest cell density-producing piezophile obtained to date, with an optimal growth at 80 MPa and 10 °C [66].

Cultivation of hyperthermophilic and piezophilic bacteria inhabiting deep-sea hydrothermal vents proved to be even more challenging, as predominant strains are often chemolithoautotrophs.

To overcome this obstacle, Takai et al. [67] developed a piezophilic cultivation technique that allows the growth of deep-sea chemolithoautotrophs, including methanogenic bacteria. The system was designed by using a combination of syringe and piston and could reach temperatures from 116 °C at 0.4 MPa to 122 °C at 20 MPa. The technique proved useful for the isolation of a new hyperthermophilic methanogenic strain i.e., *Methanopyrus kandleri* strain 116 [68]. In the follow-up work, the authors applied this system to cultivate H_2_- and/or sulfur-oxidizing chemolithoautotrophs bacteria from a thermal vent chimney. Their efforts resulted in the isolation of novel strains, one of which belongs to a novel genus of the previously uncultivated group, defined as *Thioprofundum lithotrophica*. The second bacterium was associated to a new genus of the Rhodobacteraceae family and was named *Piezobacter thermophilus* [67].

The use of specific bioreactors was also effective for the isolation of methanogenic piezophilic strains. Using a bioreactor with polyurethane sponges, the so-called down-flow hanging sponge (DHS) bioreactor, the research group of Aoki and coworkers were able to cultivate microorganisms from deep-sea sediments of 2533 m below sea surface. Methane was used as carbon sources, and the bioreactor was operated in a continuous mode. Using ^13^C-labeled methane experiments, the group was able to confirm the growth of an anaerobic oxidation of methane (AOM) community, in the bioreactor. At the end of a long incubation period (2013 days), researchers confirmed that the predominant microbial components belonged to archeal anaerobic methanotroph groups [69]. A more recent approach for the cultivation of anaerobic deep hyperthermophilic communities was performed using bacterial immobilization. In this study a mixture of microorganisms, isolated from an active thermal vent of Rainbow field at the Mid Atlantic Ridge (2275 m of depth), were immobilized into beads and used to inoculate a bioreactor in continuous mode at higher temperature (>50 °C). The culture was maintained for 45 days proving to be an effective technique for the cultivation of these microorganisms [70].

### 3.3. Metagenomics

Despite progress in improving cultivation of deep-sea microorganisms, the majority of these microorganisms remained difficult to cultivate under laboratory conditions. This also means that our capacity to obtain antibiotics from deep-sea microorganisms through traditional approaches is limited. Recent progress in DNA amplification, sequencing, and analysis has provided powerful tools to overcome these limitations. Metagenomics is a cultivation-independent technique with extraordinary potential for the study and exploitation of extreme environments [71]. This approach was successfully applied also to deep-sea environments and in the last decades it has shed some light on the biodiversity and phylogeny of bacteria, fungi, and viruses inhabiting this environment [15,72,73].

The potential of metagenomics for the discovery of natural products discovery had also been explored in the last few years. Using a functional approach, Fujita et al. were able to clone and express a biosynthetic gene cluster from a metagenomics library obtained from East China Sea sediments (1000 meter below sea surface). They generated a library in *E. coli* of about 60,000 clones with an average insert size of about 35 kbp. This library was then screened for the production of metal-binding compounds, using a chrome azurol metal ion indicator assay. In this way, a clone producing the bioactive siderophore bisucarberin was detected. The analysis of the gene clusters suggested that it was likely derived from an uncultured bacterium [74].

Using a similar approach, two metagenomic libraries were obtained using subsurface sediments collected at the depth of 3006 m in the Indian Ocean. Chen et al. [75] screened libraries for analgesic and cytotoxic activities and then selected clones which were further studied. The authors were then able to identify three indole alkaloids with promising bioactivities [75,76]. The main limitation of this metagenomics approach is related with recombinant expression. Systems based on heterologous production in *E. coli* are often inadequate to express deep-sea derived gene clusters. Thus, developing expression platforms based on piezophile microorganisms could be pivotal to increase the success rate of metagenomics. In this context, some progress has already been made. Indeed, a conjugal transfer and knock-out system have been constructed and validated for the strain *Pseudoalteromonas* SM9913, isolated from deep-sea sediment at 1855 m depth near the Okinawa Trough [77]. Yang et al. [78] have recently set up a low-temperature-inducible protein expression vector (pSW2) based on a filamentous phage (SW1) of the deep-sea bacterium *Shewanella piezotolerans* WP3. This vector can be used to successfully transform bacteria belonging to the genus Shewanella [78]. 

However, the continuous development of sequencing platforms and bioinformatics tools is making the metagenomic sequence-based approach more straightforward and effective for drug discovery purposes [79,80]. The cost of genomes and metagenomic sample sequencing is constantly decreasing, thus allowing investigations at different levels, including at large-scale. Borchet and coworkers who investigated the microbiome of three different deep-sea sponge species, collected from depths of 760 to 2900 m below sea level, using a 454 pyrosequencing for their secondary metabolomic potential, gave an example of the usefulness of this approach. They specifically targeted domains of the subunit of PKS (polyketide synthase) and NRPS (nonribosomal peptide synthase). Their data suggested the presence of many PKS and NRPS of microbial origins, indicating a huge basin of secondary metabolites [80].

Moreover, thanks to the studies of secondary metabolites biosynthetic pathways, there are many bioinformatics tools that can rapidly predict the presence of specific genes, e.g., PRISM [81], antiSMASH [82], and BAGEL [83,84]. Recently, Jackson et al. [85] sequenced the genomes of thirteen Streptomyces strains, isolated from shallow and deep-sea sponges and performed genome-mining using antiSMASH 3.0. They identified 485 clusters coding for PKS, NRPS, terpenes, and bacteriocins; the majority of which, showed little or no homology to previously reported secondary metabolite biosynthetic clusters.

## 4. Conclusions

Technologies developed over the last ten years have enabled a focus on microbial communities for the search for novel natural bioactive compounds from deep-sea habitats. Recent reports show that deep-sea microorganism-derived natural compounds may provide a new source for the development of drugs against cancer, infectious diseases, and other human ailments. 

The first step for the discovery of novel compounds should be the isolation of novel strains, which can be studied by polyphasic taxonomy. For the initial isolation, low nutrient media can be used to mimic the low nutrient content of deep-sea environments. However, it is quite possible that not all the biosynthetic gene clusters (BGC) will be expressed under laboratory conditions. Therefore, one of the most important research areas is the activation of cryptic gene clusters. Dereplication employing advanced techniques such as MS and NMR [86] improved the speed and reliability of natural product based drug discovery program. Next-generation DNA sequencing (NGS) [79] and advanced bioinformatics tools, including AntiSMASH, BAGEL, SBSPKS, and SMURF, allow the discovery and analysis of BGCs quickly. This approach, combined with new heterologous expression and pathway engineering methods, would pave a new way for the production of novel metabolites and eventually drug leads. Given the ongoing interest in deep-sea-derived natural products, it is also important to study the mechanism of actions of novel compounds discovered. Identification and manipulation of deep-sea gene clusters will make it possible for combinatorial biosynthesis to expand structural diversity. 

Increased interests in natural product drug discovery over the last decade can be accelerated via academic and biotechnology industry collaboration. Commercialization of deep-sea-derived natural products and derivatives is in its infancy but some are approaching clinical trials. Therefore, it is imperative to catalogue deep-sea habitat microorganisms for the development of new drug leads for future drugs to treat human diseases. 

## Figures and Tables

**Table 1:** A schematic summary of the new compounds with antimicrobial activity isolated from deep-sea derived microorganisms. Details are reported in the text.

	Organism	Depth	Geographic Location	Compound	Molecular Class	Antimicrobial Activity Against	Ref.
Bacteria	*Marinactinospora thermotolerans* (SCSIO 00652)	3865 m	South China Sea, People’s Republic of China	**Marthiapeptide A** (**1**)	Cyclic peptide	*M. luteus; S. aureus; B. subtilis; B. thuringiensis*	Zhou et al., 2012
	*Streptomyces scopuliridis* (SCSIO ZJ46)	3536 m	South China Sea, People’s Republic of China	**Desotamide B** (**2**)	Cyclic peptide	*S. aureus*; *S. pneumoniae;* MRSE shhs-E1	Song et al., 2014
	*Streptomyces drozdowiczii* (SCSIO 10141)	1396 m	South China Sea, People’s Republic of China	**Marfomycins A, B, E** (**3,4,5**)	Cyclic peptide	*M. luteus*	Zhou et al., 2014
	*Streptomyces* sp. (SCSIO 01127)	1350 m	South China Sea, People’s Republic of China	**Lobophorin F** (**6**)	Spirotetronate poliketides	*S. aureus; E. faecalis*	Niu et al., 2011
	*Streptomyces* sp. (12A35)	2134 m	South China Sea, People’s Republic of China	**Lobophorin H** (**7**)	Spirotetronate poliketides	*B. subtilis*	Pan et al., 2013
	*Verrucosispora* sp. (AB 18-032)	289 m	Japanese Sea	**Abyssomicin C** (**8**)	Spirotetronate poliketides	MRSA; vancomycin-resistant *S. aureus*	Bister et al., 2004
	*Streptomyces niveus* (SCSIO 3406)	3536 m	South China Sea, People’s Republic of China	**Marfuraquinocins A, C, D** (**9,10,11**)	Sesquiterpene derivative	*S.aureus;* MRSE shhs-E1	Song et al., 2013
	*Streptomyces* sp. (NTK 937)	3814 m	Saharan debris flow near the Canary Islands	**Caboxamycin** (**12**)	Alkaloid	*B. subtilis; S. lentus; S. epidermidis*	Hohmann et al., 2009
Fungi	*Emericella* sp. (SCSIO 05240)	3258 m	South China Sea, People’s Republic of China	**Emerixanthones A, B, C, D** (**13,14,15,16**)	Xanthone-derivative	*E. coli*; *K. pneumoniae; S. aureus; E. faecalis; A. baumannii; A. hydrophila*	Fredimoses et al., 2014
	*Engyodontium album (DFFSCS021)*	3739 m	South China Sea, People’s Republic of China	**Engyodontiumone H** (**17**)	Xanthone-derivative	*E. coli; B. subtilis*	Yao et al., 2014
	*Spiromastix* sp.	2869 m	South Atlantic Ocean	**Spiromastixones A, B, C, D, E, F, G, H, K, L, M, N, O** (**18,19,20,21,22,23,24,25,26,27,28,29,30,31,32**)	Depsidone analogs	*S. aureus; B. thuringiensis*; *B. subtilis;* MRSA; MRSE; *E. faecalis; E. faecium*	Niu et al., 2014
	*Penicillium* sp. (F23-2)	5080 m	Chinese Sea, People’s Republic of China	**Penicyclones A, B, C, D, E, F** (**33,34,35,36,37**)	Ambuic acid analogs	*S.aureus*	Guo et al., 2015
